# Identifying areas for action to create healthier diets in the London Borough of Newham: systems mapping with residents

**DOI:** 10.1186/s12889-025-23909-4

**Published:** 2025-08-04

**Authors:** Jessica Renzella, Adeola Agbebiyi, Ruby Nayyar, Saira Malik, Emily Ahmed, Seeromanie Harding, Peter Scarborough, Prachi Bhatnagar

**Affiliations:** 1 Nuffield Department of Primary Care Health Sciences, University of Oxford, Oxford, UK; 2 Newham Public Health, London Borough of Newham, London, UK; 3 London Borough of Newham Community Member and Newham Public Health Community Researcher, London Borough of Newham, London, UK; 4 Department of Population Health Sciences, King’s College London, London, UK; 5 National Institute for Health and Care Research (NIHR) Oxford Health Biomedical Research Centre, Oxford, UK; 6 Population Health Sciences Institute, Newcastle University, Newcastle, UK

**Keywords:** Diet, Co-design, Group model Building, Systems mapping, Interventions, London, Obesity, Diabetes

## Abstract

**Background:**

Healthy diet is an essential component of good health, yet many areas of the UK struggle with high burdens of diet-related diseases. Efforts to address diet-related diseases in the London Borough of Newham have had limited success so far, possibly due to the lack of engagement with Newham’s distinct local context. Newham is ethnically diverse and within the 20% most deprived areas of England. To engage residents in public health action and encourage approaches that tackle the underlying causes of poor-quality diets, systems approaches were used to co-design healthy diet interventions with residents in Newham. The specific aims of the project described in this paper were to understand residents’ perceptions of the determinants of unhealthy diets and identify their desired areas for action.

**Methods:**

Twelve online Group Model Building workshops were conducted with 33 Newham residents from six Community Neighbourhoods. Participants reflected the ethnic and religious diversity of Newham’s population. The first workshop explored residents’ views of what is causing people to have unhealthy diets. Participants identified areas for action and brainstormed solutions to improve diets in the second workshop.

**Results:**

Each workshop produced a neighbourhood-specific systems map of ‘what’s causing people in Newham to have unhealthy diets’. Residents identified multiple and connected political and economic, physical environment, social environment, and individual level causes of unhealthy diets. Suggested action included increasing food and nutrition education, addressing the unhealthy influence of social media, alleviating poverty and improving food business practices.

**Conclusions:**

Online Group Model Building activities represent a comprehensive yet low cost and low burden method for engaging communities in identifying areas for action to improve diets. The systems maps created in this project with Newham residents have been used to co-develop context-specific food interventions with Newham Council that focus on improving the food environment.

**Supplementary Information:**

The online version contains supplementary material available at 10.1186/s12889-025-23909-4.

## Introduction

Suboptimal diet is one of the leading causes of disease in the UK, with some populations more at risk than others [1]. According to the World Health Organization, an optimal diet for adults includes: a balance between energy intake and energy expenditure; limiting intake of salt and sugar; increasing consumption of fruit, vegetables, legumes, nuts and whole grains; limiting consumption of fats to less than 30% of total energy intake; shifting fat consumption away from saturated fats; and eliminating trans-fats [2]. Those most at risk of diet-related ill health include people who are on lower incomes, live in deprived areas, and are from minority ethnic backgrounds [3, 4]. Effectively and equitably changing diets can have a significant impact on population health and reduce inequalities in health outcomes [5, 6].

The London borough of Newham is a highly diverse area: 73% of its population is from an ethnic minority background; 48% of residents were born outside of the UK; and over 100 languages are spoken [7, 8]. In 2021, 42.2% of people in Newham identified their ethnic group as ‘Asian, Asian British or Asian Welsh’, 17.5% as ‘Black, Black British, Black Welsh, Caribbean or African’, and 4.9% as ‘Other’ (‘Arab’ or ‘Any other ethnic group’) [8]. Newham is also within the 20% most deprived areas of England and has high rates of overweight and obesity (63%) and type-2 diabetes (8.6%) when compared to London overall. When ethnically-adjusted body mass index cut-offs are used, the prevalence of overweight and obesity rises to 70% and type-2 diabetes is up to six times more likely in South Asian populations and up to three times more likely in Black Caribbean and Black African groups [9, 10]. To address diet-related ill-health in Newham, the council has implemented a variety of preventative measures, with the majority aimed at individuals [10]. However, in their Health and Wellbeing Strategy (Well Newham 50 Steps to a Healthier Borough), Newham Council describe how high levels of health inequalities, spotlighted during the COVID-19 pandemic, necessitate investment in more ambitious measures if they are to effectively reduce health inequities [10, 11].

Reducing inequalities requires an understanding of how different people experience interventions and their benefits differently. The level of agency required for individuals to benefit from an intervention impacts how and for whom interventions work. For example, prevention programmes that focus on individual-level behaviour change, such as dietary counselling whose success relies on individuals engaging with information, may not have universal benefits and may in fact widen inequalities [12]. This is demonstrated by a recent evaluation of a national diabetes prevention programme that reported fewer intervention benefits (e.g. weight loss) associated with Asian and Black ethnicity, lower socioeconomic circumstances, and baseline overweight and obesity [13]. Such programmes are also likely to struggle to achieve lasting change even in groups who do engage with the programme, as they do not address the root causes of conditions like diabetes that are driving people towards unhealthy diets. Tackling the determinants of unhealthy diets is, however, a complex task. Multiple interconnected factors impact our food system and interaction with it, and contextual factors mean that solutions that work in one context might be less successful in another [14]. Further, many food system challenges that existed before the pandemic have been exacerbated since. The cost of living crisis, for example, has seen record increases in the cost of food and energy, disproportionately impacting people on lower incomes [15].

To ensure diet-related interventions are proportionate, appropriate, and acceptable, it is important to understand the system that influences diet as experienced by the people who live in the context of interest/intervention. Through supporting the visualisation of complex problems and identifying the wider determinants of unhealthy diets, systems mapping methods can help residents articulate the wider influences on their diet and prioritise which are the most important to address [16]. Group Model Building (GMB) is a participatory research approach that can help communities to develop systems maps by encouraging collective consideration of problems and potential solutions [17]. This method, which has been used with diverse populations in different countries, can be especially helpful for co-creating upstream measures for preventing unhealthy diets [18, 19].

This paper presents the findings from the first phase of a wider, multicomponent project which aims to co-develop a context-specific, feasible, and effective population-level intervention to improve diets in Newham, London.

## Methods

The aims of the project described in this paper were to understand residents’ perceptions of the determinants of unhealthy diets and identify their desired areas for action. GMB workshops facilitated the creation of modified Causal Loop Diagrams (CLDs) to achieve project aims. A CLD is a visual representation of a system where system variables (text) are connected by arrows that show the causal relationships between them [20]. The GMB methods used in this study (including workshop activities and prompts) were informed by [17].

### Research team

The core research team was comprised of two academics from the University of Oxford (JR and PB), with experience in workshop facilitation and application of qualitative research methods, and two Community Researchers (RN and SM). Community Researchers, who are Newham residents with an interest in research and healthy diet promotion, supported the development of workshop materials, participant recruitment, and workshop observation/notetaking. Community Researchers were recruited and trained by the Newham Public Health Peer Researcher Programme manager who is a Public Involvement specialist (EA). The Peer Researcher Programme supports community members to carry out their own research projects as well as support other projects and insight gathering activities within Newham Public Health. The programme values lived experiences and aims to challenge traditional researcher and participant relationships, minimise power imbalances, and build trust and capacity within the community.

### Setting

It is usual for GMB activities to take place in person [21], however, due to COVID-19 restrictions and concerns, workshops were hosted on an online meeting platform (Zoom) and GMB methods were adapted accordingly. Workshop participants used their personal devices (phones, computers, tablets) to connect to Zoom meetings; participation was not restricted to those with a laptop or computer in order to keep the research accessible to all. To ensure the cost of joining was not a barrier to participation, all participants were eligible for mobile data cards. In response to council project partner recommendations, workshops were designed to accommodate a wide range of participant education levels, digital literacy, and English language proficiency. For example, each workshop activity was explained using accessible language that had been approved by resident community-researchers in a pilot exercise, all activities were demonstrated by the facilitator before participants were asked to complete a task, and paper-based activities were prioritised over tech-based engagement.

### Recruitment

Participants were recruited from Newham’s eight Community Neighbourhoods (East Ham, Beckton & Royal Docks, Custom House & Canning Town, Green Street, Plaistow, Manor Park, Forest Gate, Stratford & West Ham) via email, social media, and word of mouth. The decision to target recruitment by neighbourhood was twofold: (1) Newham Council recommended an inclusive approach to recruitment that did not exclude certain groups and (2) neighbourhood-specific recruitment allows for the exploration of specific issues in different areas characterised by different levels of income deprivation, types of housing and living arrangements, and other population sociodemographic factors such as educational attainment and employment status [7, 22]. Community organisations and council networks were also contacted via email to help amplify recruitment through their newsletters, email lists, and other communication channels (e.g. Facebook and WhatsApp). Over 45 community groups and organisations were contacted in an attempt to reach Newham’s diverse population. Participants had to be at least 18 years old, a Newham resident, and comfortable communicating in English. Due to the online nature of workshops, the research team aimed to recruit a minimum of four and maximum of 10 participants per workshop to help ensure high quality of facilitation and high levels of participant engagement. Participants provided informed consent using an online form and received a £20 voucher for each of the two workshops they participated in.

### Workshops

The research team ran two Zoom workshops per neighbourhood between June and August 2022. Workshops were held at a time of day that was suitable for participants. For this reason, most workshops took place in the evening. Each workshop was approximately three hours in duration and facilitated by JR. PB supported workshop activities and RN and SM took notes. Workshops followed a structured format based on the GMB process outlined by [17]. Multiple comfort breaks were scheduled during each workshop and JR emphasised that participants were able to leave the workshop or their device at any point without having to provide a reason for doing so. All workshops were recorded for notetaking purposes.

The first workshop (Table 1) explored residents’ views of what they believe is causing people to have unhealthy diets. Before the workshop participants were mailed ‘participant packs’ which included a cover letter, participant information sheet, workshop worksheets, data card (if requested), pen, and paper.

**Table 1:** Description of workshop 1: exploring residents’ views of what they believe is causing people to have unhealthy diets

Activity	Description
Introductions and icebreaker	Participants were invited to introduce themselves and asked why they wanted to join the workshops and what they hoped to get out of them.
Housekeeping	Not all participants had experience using Zoom so key Zoom functions and etiquette were described. This time was also used to check participants had received their participant packs and had access to a camera phone for capturing workshop outputs.
Project overview and introduction to systems thinking	Participants were introduced to the project context and intended use of outcomes, rationale for conducting two workshops, and thinking about the food we eat as part of a system.
Activity 1: Listing exercise	Participants were asked ‘why do people in your neighbourhood eat unhealthy food or have unhealthy diets?’ PB shared her screen and made a list of their responses in a Word document. Participants were asked to elaborate on their responses. Discussion between participants was encouraged.
Activity 2: Graphs over time	Each participant was tasked with choosing one cause from the list created in Activity 1. Participants were then asked ‘how has this changed over time?’ The graphs over time template (Fig. 1), with time on the X-axis and chosen variable on the Y-axis, helped participants share their perception of how a particular cause of unhealthy diets had changed in their community over time. JR provided a step-by-step demonstration of how to complete the worksheet before participants were given time to complete their own change over time story. Participants were encouraged to think about the present (‘what’s the situation today?’) first, before thinking about the past and then the future. When thinking about the future, participants were asked to draw both their hopes (‘what might the future look like in your ideal world?’) and fears (‘what might the future look like if positive change doesn’t occur?’). Participants were then asked to share their change over time story with the group.
Activity 3: Cognitive mapping	In Activity 3, participants were asked ‘what are the causes and effects of unhealthy diets and how are they connected?’ They were provided with a cognitive map template (Fig. 2) to help them describe and connect the causes and consequences of unhealthy diets. For each quadrant of the template, JR provided an example before asking participants to complete the quadrant. Participants were invited to tell the group about important or interesting connections that they drew.
Data collection	At the end of the workshop, participants were asked to take a photo of each completed worksheet and upload images to a secure University of Oxford data sharing website. The link for this website was sent to participants via text message.
Workshop close	JR summarised the workshop and introduced the aims of Workshop 2. The research team thanked participants for their time and contributions.

**Fig. 1:**
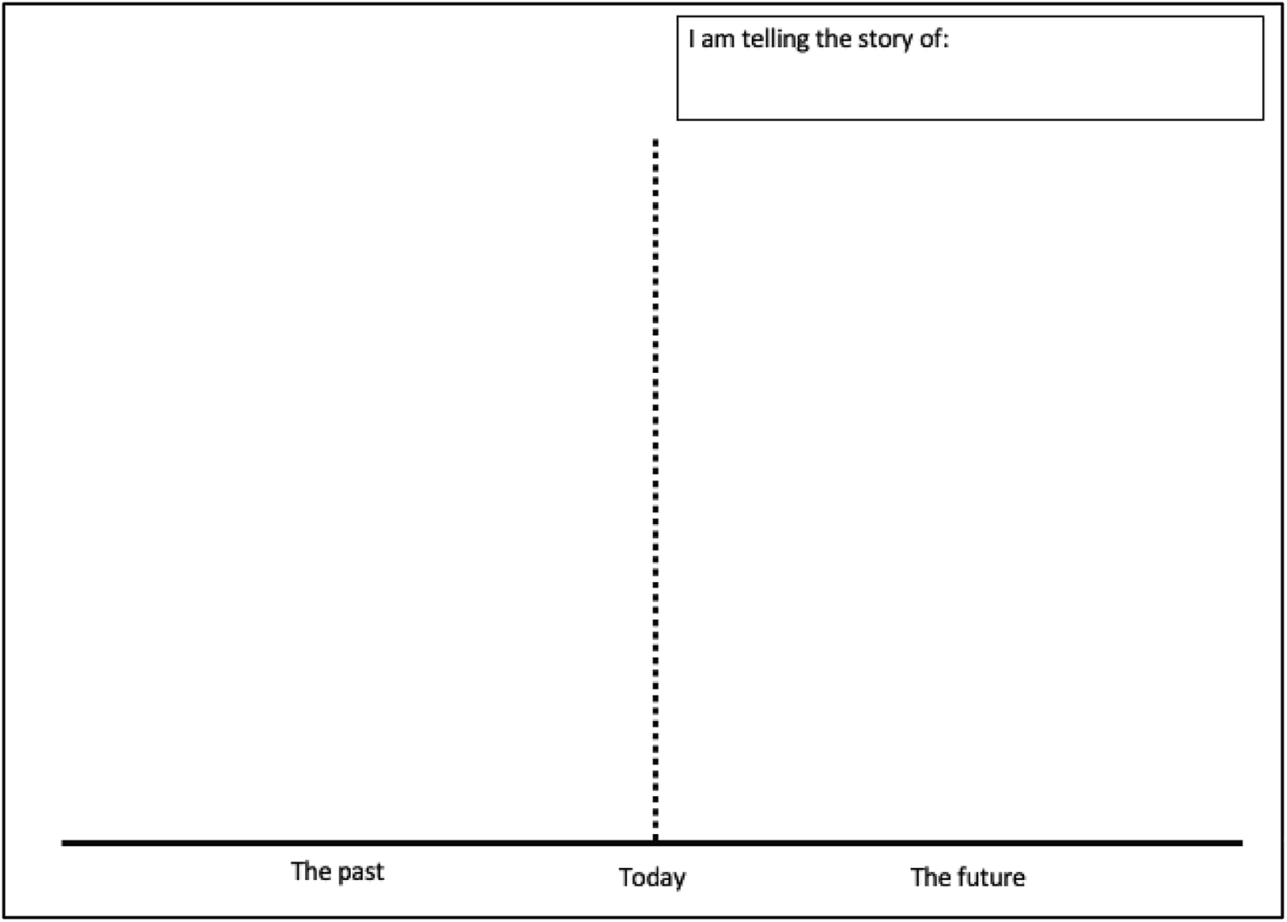
Graphs over time template adapted from [17]

**Fig. 2:**
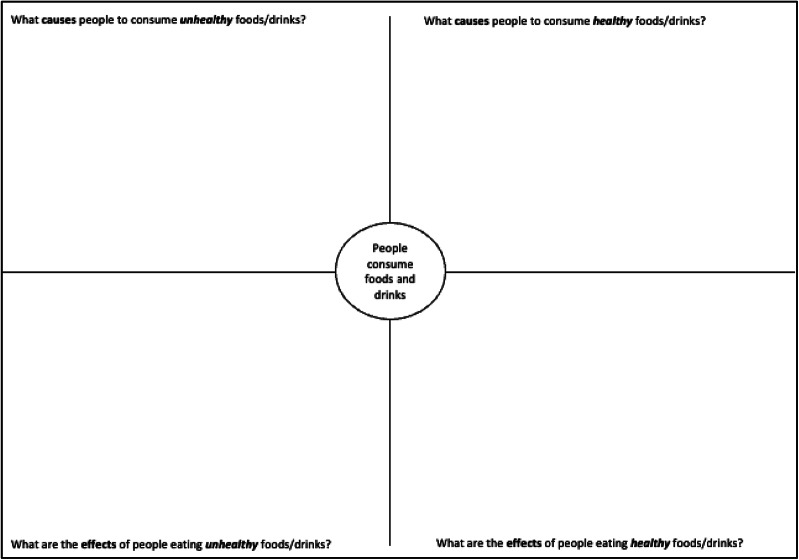
Cognitive mapping template adapted from [17]

To account for the fact that many participants joined the workshops using phones and were therefore unable to use mapping software, between the two workshops JR and PB created neighbourhood-specific CLDs from participants’ cognitive maps and workshop notes in Vensim simulation software. The CLDs created were simple CLDs that did not include polarity (the type of relationship that exists between two variables represented by ‘+’ and ‘-’ symbols) and were referred to as ‘systems maps’ during workshops. Once created and reviewed, maps were themed and colour-coded to facilitate explanation to the workshop participants.

The second workshop (Table 2) was held 1–2 weeks after Workshop 1. In the second workshop, participants reviewed the CLD, identified areas for action, and brainstormed solutions to improve diets in their neighbourhoods. Brainstormed solutions were transcribed and grouped by identified areas for action.

**Table 2:** Description of workshop 2: map review and identification of action areas and solutions

Activity	Description
Introductions and icebreaker	Participants were asked to re-introduce themselves and share one thing they enjoy about living in their neighbourhood.
Project and Workshop 1 review	Participants were reminded about the aim and structure of the wider research project and about Workshop 1 activities and outcomes.
Thinking about the food we eat as part of a connected system review	Participants were reminded about ‘thinking in systems’.
Activity 1: Map review	Participants were introduced to their ‘map of what’s causing unhealthy diets in my neighbourhood’. PB shared her screen to display the map in Vensim. Theme by theme, JR talked through each variable and connection and asked participants • Is there anything you would like to add to the map? • Is there anything you would like to change about the map? • Did we get the connections right? PB made live changes based on participants’ verbal feedback.
Activity 2: Identifying areas for change	Participants were asked, ‘if you had to pick one colour area on the map to change, which would it be? Each participant was provided an opportunity to select an area and share the reasons why they had selected it. PB shared Vensim again and JR asked participants to select specific action sites (map variables) within their chosen areas that they thought were particularly important to focus on. Participants were not restricted in number of selections. Upon selection, PB highlighted the variable(s) of interest in a different colour.
Activity 3: Brainstorming solutions	Finally, participants were asked ‘what interventions would you like to see implemented to address the issues we’ve identified?’ Participants were encouraged towards ‘blue sky’ thinking and given time to independently make a ‘list of solutions’ before feeding back to the group.
Workshop close	JR summarised the workshop and discussed next steps for the project. The research team thanked participants for their time and contributions.

### Ethics

Ethics approval for the study was obtained from the University of Oxford Central University Research Ethics Committee (CUREC) (ref R74296/RE001).

## Results

### Participants

The workshops included 33 Newham residents from six Community Neighbourhoods: Stratford & West Ham, Manor Park, Forest Gate & Maryland, East Ham, Plaistow, and Beckton & Royal Docks. Not enough participants (fewer than three) were recruited from Green Street and Custom House & Canning Town to run workshops. One participant who was unable to join Workshop 1 participated in Workshop 2 only. Participants reflected the ethnic and religious diversity of Newham’s population (Table 3).

**Table 3:** Partcipant characteristics

Participant characteristics, self defined	Number of participants (*N* = 33)
**Participant gender**	
Female	27
Male	6
**Participant age**	
18–34	10
35–54	16
55–74	6
Prefer not to say	1
**Participant ethnic group**	
Any other Black/African/Caribbean background	1
Any other Mixed/Multiple ethnic background	1
Any other White background	2
Asian/Asian British: Bangladeshi	3
Asian/Asian British: Indian	7
Asian/Asian British: Pakistani	5
Black/African/Caribbean/Black British: African	3
Black/African/Caribbean/Black British: Caribbean	3
Mixed/Multiple ethnic groups: White and Black Caribbean	1
Prefer not to say	2
White: English/Welsh/Scottish/Northern Irish/British	5
**Participant religion**	
Christian	11
Hindu	3
Muslim	12
No religion	2
Prefer not to say	3
Sikh	1
Spiritual	1
**Neighbourhood**	
Beckton & Royal Docks	3
East Ham	6
Forest Gate	5
Manor Park	8
Plaistow	5
Stratford	6

### What do residents feel is causing unhealthy diets in Newham?

**Fig. 3:**
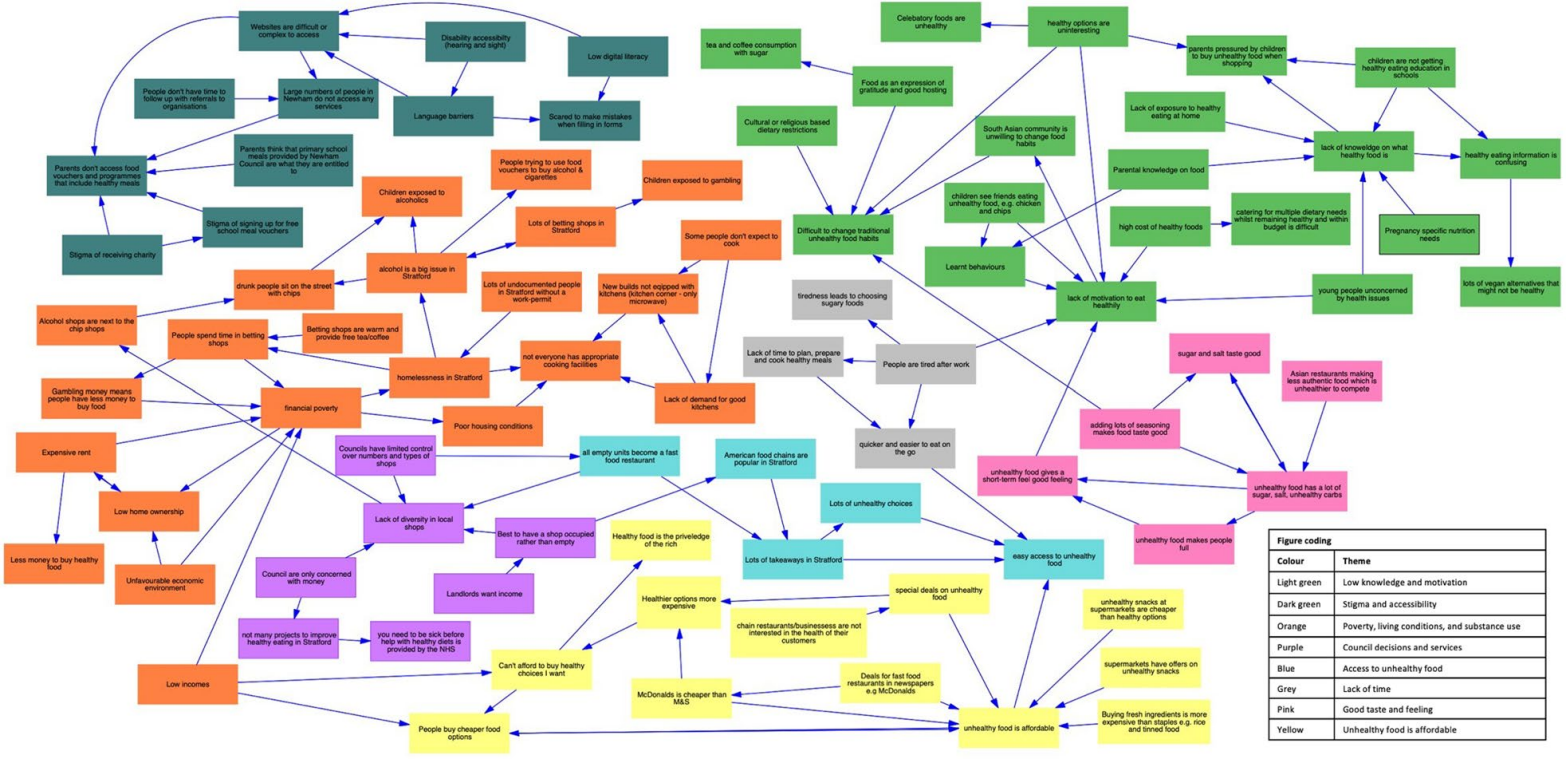
Neighbourhood-specific systems map created during Workshop 1 of ‘what’s causing people in Stratford to have unhealthy diets’

Each set of workshops produced a neighbourhood-specific systems map of ‘what’s causing people in Newham to have unhealthy diets’. Maps supported residents to identify problems and solutions, and allowed researchers to communicate these effectively with stakeholders in the second phase of the project that focused on intervention development (not described in this paper). Systems maps were created in Workshop 1 with all variables reviewed at the beginning of Workshop 2. This allowed participants to refine and add variables. Figure 3 provides an example of the type of map produced by residents.

There was a high degree of homogeneity between map themes, with only two neighbourhood maps identifying unique themes that were not identified in others: ‘Council decisions and services’ was specific to Stratford & West Ham and ‘Price and taste of [London] water’ was specific to Beckton & Royal Docks. The themes identified by Newham residents as causing unhealthy diets in the area included political and economic, physical environment, social environment, and individual level causes (Table 4).

**Table 4:** Themes identified by Newham residents as causing unhealthy diets in the area, grouped by type of influence

Type of influence	Themes identified by Newham residents as causing unhealthy diets in the area
Political and economic causes	• Council decisions and services • Lack of education, information, resources • Financial issues, poverty
Physical environment causes	• Living conditions • Food access, availability, affordability • Price and taste of London water
Social environment causes	• Food marketing • Social influences • Food culture and eating behaviour • Stigma and accessibility
Individual-level causes	• Lack of food and nutrition knowledge • Lack of time • Lack of cooking skills and resources • Mental health, emotions, and substance use • Food taste and emotional response • Lack of motivation

### Areas for action

Participants identified six themes across the six system maps where they wanted to see change (areas for action), and 25 specific variables within those areas for action that they believed needed to change in order to improve diets in their local area (Table 5). Some participants selected more than one variable as they felt that only changing one would not be sufficient.

**Table 5:** Causes of unhealthy diets that participants wanted to address

Identified areas for action	Specific map variables identified as needing to change
Council decisions and services	• Councils not incentivising high street diversity. • Lots of licenses given to similar unhealthy food stores that are next to each other. • No borough-wide programme for healthy eating in schools. • Not enough support for creative business solutions. • Residents do not have a say about what local shops can open.
Food access, availability, affordability	• Unhealthy food is cheaper than healthy food. • Cost of food is rising. • Unhealthy food is everywhere and easier to access than healthy food. • No variety – lots of chicken and chip shops. • People don’t need to cook when there are so many food delivery apps. • Lack of offers on healthy food.
Lack of food and nutrition knowledge	• People unsure what constitutes healthy food. • Lack of knowledge about unhealthy food. • Lack of easily accessible information about healthy food/eating. • Lack of knowledge of how to do a healthy food shop on a budget.
Food marketing	• People are bombarded with unhealthy food advertising. • Subliminal food advertising on social media. • Young people receive mixed messages from social media. • Large banners with unhealthy food advertising and promotions.
Financial issues, poverty and living conditions	• Lots of poverty in Newham. • The basics of food and shelter are not always provided in Newham. • Lack of rent control.
Mental health and emotions	• Poor mental health is linked to unhealthy eating. • Concerns over body image. • Newham does not feel like a cohesive community (no Newham pride, apathy).

### Intervention ideas

After selecting areas for action, residents suggested interventions that in an ideal world (for example, one where local council budget restraints would not preclude the implementation of more expensive interventions) they would like to see implemented to improved diets in Newham. Participants suggested many different types of interventions, some of which provided further insight into the problem which led to the rewording and/or addition of map variables. Participants were not limited to suggesting a certain number of interventions and so 62 solutions were shared. Examples from a range of participants, categorised by action area, included:

### Council decisions and services

*“Discounted business rates for people who sell healthier options to the community” (Stratford participant).*

*“Council should engage with independent fast-food outlets and encourage them to shift to selling healthier foods…for those who have the autonomy to make changes*,* council can engage with them to provide positive training opportunities*,* networks*,* and practical guidance of how to provide healthy options.” (Beckton & Royal Docks participant)*.

*“More planning controls. Reduce the number and density of fast-food outlets around workplaces*,* colleges*,* etc.” (Beckton & Royal Docks participant)*.

*“All food that can be influenced by council*,* for example in council offices*,* schools*,* leisure centers*,* should only offer healthy food at accessible prices.” (East Ham participant)*.

### Food access, availability, affordability

*“Government taxes on unhealthy food.” (Manor Park participant)*.

*“Supermarkets already have promotions on fruit and veg*,* but these aren’t consistent. They go on and off. [These promotions/lower prices] should be mandatory and consistent every day*,* all year.” (Manor Park participant)*.

*“Pop up grocers [supported by the local authority] can encourage people to try a new veg*,* raw or cooked in a meal*,* and if you like it*,* it’s available to buy there and then. Expose people to something new*,* different*,* healthy*,* and delicious.” (East Ham participant)*.

### Lack of food and nutrition knowledge

*“Hands on workshops with community members…to teach healthy eating and cooking… [opportunity to] share a community meal at the end.” (Beckton & Royal Docks participant)*.

*“Access to online courses for parents that are short…make it obvious where they are*,* for example*,* signpost these in nursery and school newsletters. These have to be engaging not just a library of information people need to read. Not patronising. Not intimidating.” (Plaistow participant)*.

*“Provide education but school meals should also match this education…in secondary school nutrition and cooking lessons should be part of the core curriculum for all students.” (Manor Park participant)*.

### Food marketing

*“Unhealthy food warnings…like for smoking.” (Forest Gate participant)*.

*“Ban the use of cartoon characters to advertise unhealthy food [to children].” (East Ham participant)*.

*“Positive advertising for healthy food and lifestyles…on buses and in public places…so always in the back of people’s minds. (Manor Park participant)*

*“Hold big food companies to account in terms of forcing them to be more responsible and ethical with advertising…[for example] ban fast food advertising during children’s programmes on YouTube.” (Beckton & Royal Docks participant)*.

### Mental health and emotions

*“Improve mental health services through training to encourage reference to healthy eating as part of holistic approach.” (Beckton & Royal Docks participant)*.

A full list of suggested intervention ideas is included in Additional File 1.

## Discussion

This paper describes GMB workshops conducted with six groups of Newham residents to understand their perceptions of the determinants of unhealthy diet as well as their desired areas for action. Across the six groups of residents, grouped by Community Neighbourhood, there was little difference in the factors they identified as influencing unhealthy diets in their area. This finding suggests that diverse groups in Newham experience similar unhealthy food environments. For Newham Council, this means that although the implementation of interventions in different areas may require tailoring, the focus of interventions to tackle the determinants of unhealthy diets could be similar across neighbourhoods. To address mapped risk factors, participants identified 25 specific areas for action ranging from council policy action to improving the perceived widespread lack of food and nutrition knowledge. These results suggest that multilevel complex interventions might be needed to address individual-level and environmental-level risk factors. Evidence from other studies have demonstrated the positive impacts that these types of interventions can have in low-income ethnically diverse areas [23]. Participants also shared intervention ideas that offer insight into the types of interventions residents believe will work to improve diets and possibly the types of interventions they would find acceptable. For example, workshops revealed public support for tackling environmental-level risk factors. The systems maps, prioritised factors, and suggested solutions reported here formed the basis of further intervention co-design work with Newham Council, local businesses, and other food stakeholders. Newham Council’s current approach (proposed and implemented) to addressing unhealthy diets does include both individual and environmental change, such as its approach to addressing food insecurity among young people [24]. However, a dearth of intervention evaluation data makes it difficult to assess the success of current approaches, and plans (outlined in strategy documents) do not always translate to action for financial, logistical, political, and other reasons [25]. Projects like the one outlined in this paper can aid the intervention development and implementation process.

This study takes a similar approach to others that have also used GMB with specific communities or groups of people to understand the determinants of diet and obesity better so as to develop effective, appropriate, and innovative interventions [17, 18, 26]. Key differences between many of these papers and this one include the use of in-person facilitation and the production of complex CLDs. The decision to conduct online workshops, and consequently construct simplified diagrams, was due to concerns surrounding the COVID-19 pandemic. Although tools are available to support the creation of complex CLDs with participants in virtual settings, the online approach taken in this study was tailored to participants who were encountering systems thinking for the first time, whose first language was not necessarily English, who may have low digital literacy, and who were joining workshops on their phones. CLDs were therefore simplified in this study to illustrate variables, connections, and themes only.

In terms of map outputs, it is difficult to compare directly those produced in this study with others due to the context-specific nature of such studies (participant characteristics and experiences, geographical location, political context, research questions, data collection method, analysis framework, etc.). However, comparisons can be drawn that build a compelling case for focusing prevention efforts on population-level risk factors. In a GMB exercise conducted with adolescents in European countries to identify the key drivers of obesity, recurring themes included: advertising, low cost and easy access of unhealthy foods, social media, low physical activity, and mental health factors (e.g. stress and body image) [27]. An older study conducted in Australia shows that the fast food and junk food environment as well as social influences (which included school, community, and workplace factors) influence dietary behaviors [18], and a study conducted in Amsterdam identified that profit maximisation (in the food and tech sector) as well as a focus on individual responsibility drive obesity in adolescents [28]. All of these themes were identified by Newham residents, and all can be tackled by environmental change.

### Strengths and limitations

The strengths of this study include the GMB approach used and its adaptation to an online format. The GMB approach allowed participants to build towards thinking in systems. Structured activities provided multiple opportunities for discussion, reflection, and for participants to express themselves in different ways (e.g. exchanging views through discussion, drawing pictures, storytelling). The method also allowed participants to share their views in general terms, which may have helped them to feel more comfortable sharing potentially sensitive information about weight, mental health, and financial issues. This GMB strength has also been identified by Savona et al. who worked with adolescents across Europe on the topic of obesity [26]. Many participants shared that they enjoyed that workshops were remote: some joined while taking care of children and family members and some while commuting home from work. Some participants also took breaks from their screen to pray, answer the door for deliveries, or check on family members. Virtual workshops therefore allowed participants who may not have been able to attend in-person sessions to contribute to the project. Zoom workshops were also cheaper to run and easier to organise than in-person workshops, although this was not the reason why this method was selected. Similar positive experiences were reported by Chavez-Ugalde et al., who ran online GMB workshops on the commercial determinants of dietary behaviours in adolescents: “adapting, and delivering GMB online is feasible, engaging, cost-saving and an enjoyable experience” [29]. GMB with local residents produced rich data that revealed contextualised determinants of unhealthy diets that may not have otherwise been uncovered if residents were not engaged in this participatory manner. For example, specific grievances with local council actions, the stigma that can surround receiving support, and the high cost of culturally-specific foods were not factors that had been discussed with researchers outside of workshops. Participants indicated desires for change that extended beyond the remit of this project, and that could therefore not be addressed in further stages of the project. Although data were not collected on participants’ experiences, many participants made a point of thanking the research team for the opportunity to engage in stimulating conversations and activities about food and health in Newham that were solutions focused.

Although one of the key strengths of workshops was their online nature, digital exclusion – where an individual lacks the necessary knowledge and access to internet/online meeting platforms to be able to fully participate in activities [30] – may have meant that some people were not able to join the session. The research team did try to mitigate the effects of digital exclusion by paying for access to internet for participants who requested support, and participants were able to join on any type of device with access to the internet. Two researchers created neighbourhood-specific CLDs from participants’ cognitive maps and workshop notes. To overcome potential limitations that might be associated with this approach, for example, incorrect interpretation of map variables and connections, maps were reviewed in detail during the second workshop and participants were invited to amend and extend maps. There is of course more diversity in the community than the workshops described in this paper were able to capture. Workshops were conducted in English due to resource constraints and this may have limited the diversity of views and voices captured by the process. More women signed up to participate than men and so more women were included in this study. Previous research suggests that gender-based willingness to participate in voluntary research is not uncommon and may be due to a variety of factors including the fact that women are more likely to participate in community-based activities [31, 32]. Additionally, the official demographic categories used for recruitment, although useful, are wide and as a result may not capture diversity within those groups. Workshops were not hosted in Green Street and Custom House & Canning Town neighbourhoods as sign-up numbers were too low, but the Green Street area (a destination for South Asian shopping and eateries) was the focus of many workshop discussions. Finally, GMB findings are limited to the perceptions of workshop participants which is why the workshops form one part of a larger programme of work that includes reviews of the literature and consultation with other stakeholders.

### Researcher reflections

A key overarching strength of this study is that it was born from a partnership with local council who are responsible for service provision and policy change (within the boundaries of local government). Support from Newham Council was imperative for developing and running the project and ensuring that workshop outcomes had a home, which was very important to participants. Developing a strong relationship with members of the council also strengthened recruitment efforts. The involvement of Community Researchers was a great benefit to the project. Community Researchers provided helpful feedback during the pilot process and their workshop notes and reflections helped Oxford researchers to understand the Newham context better. Their participation also contributed to their upskilling at an early stage of their research careers. Participatory research with ‘hard to reach’ communities is imperative but can come with administrative challenges that should not dissuade researchers but rather encourage academic institutions to better facilitate such work. For example, the research team found it difficult at times to engage with community members in the way they had requested (for example, using WhatsApp rather than emails) while also satisfying institutional ethical and information governance requirements. Reconciling these differences extended the time required for approvals which at times delayed the project.

### Further research

It might be helpful to investigate whether broader consultation would extend paper findings and detect more nuanced results for different communities. For example, additional workshops could be hosted in the Green Street and Custom House & Canning Town neighbourhoods, with more diverse adult participants (e.g. participants who may have been excluded due to language barriers), and with different population groups (e.g. school children in Newham). Hosting workshops in languages other than English may also reveal more barriers relating to the culture of participants, as well as the wider environment. The creation of more complex maps that can support a systems evaluation of interventions could also be the focus of future work. Future GMB projects should consider embedding participant feedback within the workshop process. Consultation with Newham Council is essential for guiding any further research in this area as the research environment in the borough is active and dynamic. Reviewing ‘Well Newham − 50 Steps to a Healthier Borough: Health and Wellbeing Strategy 2020–2023’ may also help guide further research [11].

## Conclusion

Online GMB activities represent a comprehensive yet low cost and low burden method for engaging communities in identifying areas for action to improve diets. Participants identified a wide range of factors, from individual-level influences to environmental and political influences, that contribute to unhealthy diets in their neighbourhood. Some of the issues identified by participants are already being addressed by the council and others were beyond the scope of this project. The systems maps created in this project with Newham residents have been used in further research to co-develop a context-specific intervention with Newham Council that aims to improve the food environment by focusing on local businesses and the healthiness of food provision.

## Electronic supplementary material

Below is the link to the electronic supplementary material.

Additional file 1: Intervention ideas suggested by participants during Workshop 2, by neighbourhood.

## Data Availability

The data generated and analysed during the current study are not publicly available as ethical approval does not extend to the sharing of participant data or workshop recordings beyond study authors.

## Abbreviations

CLD: Causal Loop Diagram
GMB: Group Model Building
