# Supplementary material for: Identifying areas for action to create healthier diets in the London Borough of Newham: systems mapping with residents
Source: BMC Public Health. 2025 Aug 4;25:2645. doi: 10.1186/s12889-025-23909-4 (PMC12323162; doi:10.1186/s12889-025-23909-4)
Supplement: Supplementary file 1 — Additional file 1: Intervention ideas suggested by participants during Workshop 2, by neighbourhood. [file 12889_2025_23909_MOESM1_ESM.pdf]

Additional Files

Additional File 1. Intervention ideas suggested by participants during Workshop 2, by neighbourhood

Neighbourhood	Suggested intervention ideas
**Beckton & Royal Docks**	• Improved mental health services and training on healthy eating as part of holistic approach.<br>• Commercial role modelling: network of shops that only have healthy food.<br>• Council engagement with/training for independent fast-food outlets to encourage shift to selling healthy food.<br>• Planning controls to reduce number, density, proximity of unhealthy food outlets close to workplaces, colleges, etc.<br>• Incentives for healthy restaurants, for example, altered business rates, cheap rentals, free training, free equipment.<br>• Networking with other businesses in other boroughs doing better.<br>• Annual healthy food festival to celebrate healthy food and food in Newham.<br>• Hands-on, engaging workshops with community members (separate for children and adults) to teach healthy eating. Could include sharing a meal.<br>• All restaurants add healthy food options to the menu e.g. salads.<br>• Better use and redistribution of food: focus on reducing food waste by reusing and sharing within the community so that no-one goes hungry.

East Ham	• Close fast-food outlets within close proximity to schools.<br>• All food provided/influenced by council (e.g. council offices, schools, leisure centers) should be healthy and affordable.<br>• Co-operative style not-for-profit market stalls that provide healthy food at cost (will need to be subsidised).<br>• Hold big food companies to account in terms of forcing them to be more responsible and ethical with advertising. This includes banning fast-food advertising during children's television programmes and children's programmes on YouTube.<br>• Ban the use of cartoon characters to advertise unhealthy food.<br>• Food surgeries/ask the expert days that anyone can access (like council surgeries).<br>• Gyms in borough can offer more nutritional advice (if people want it).<br>• Local authority can support pop-up grocers where people can try a vegetable (raw or cooked in a meal). If they like it, then it is available to buy.<br>• Health champions in schools. (These might be children or experts).<br>• Set up an example healthy chicken shop to encourage other chicken shops to add some healthier options.<br>• Hold influencers more accountable for the fast-food they promote online, maybe through regulation.<br>• Organise a campaign for local celebrities to promote healthy eating.
Forest Gate	• More free activities for young people and families in Newham where people can find information, engage in activities, and have discussions with nutrition experts.<br>• Stop offers on unhealthy food during peak times when children buy unhealthy foods.<br>• After school clubs that meet more often and focus on healthy food and activity. Make it convenient and close to schools.<br>• GP follow up appointments for healthy eating that provide practical tips.<br>• Advertising for healthy foods and lifestyles.<br>• Close fast-food outlets around schools for an hour after school ends. Promote other social spaces.<br>• Unhealthy food warning advertisements (like anti-smoking adverts) on public transport and in public places.<br>• Heavy tax on processed food so that shops that sell more wholefoods pay less and those that sell unhealthy highly processed foods pay more.

**Manor Park**	• Supermarket promotions on fruit and vegetables should be mandatory and consistent every day of the year.<br>• Short courses on healthy eating, nutrition, cooking open to everyone with a certificate/certification.<br>• Healthy eating workshops for children in schools.<br>• More education and workshops for pregnant people and school children (part of mandatory curriculum). School meals should be healthy to match healthy eating education.<br>• Educate parents not just on healthy foods and cooking but how to deal with children who demand unhealthy food.<br>• Government taxes on unhealthy foods where tax passed onto consumer, so these foods are more expensive and people will not want to buy.<br>• Positive advertising, leaflets, brochures, posters so healthy eating always on people's minds.
**Plaistow**	• Limit leases for fast-food restaurants.<br>• Cash incentives for healthy fast-food business (e.g. cash grant if in business for X years).<br>• Rates relief for healthy businesses.<br>• Store fit out funds for healthy food businesses.<br>• Practical visits with nutritionists in children's centres and schools.<br>• Accessible, empowering, short, reputable, and well-sign-posted online courses for parents.<br>• More council action on regulating and incentivising healthy food businesses.

**Stratford**	• Regulate unhealthy food advertising on social media, especially targeted to young people.<br>• Local festivals and events that include food provisions must include healthy eating options.<br>• Supermarket pricing strategy should ensure that healthy snacks are cheaper than the unhealthy alternatives.<br>• Council should implement a scoring system to score shops as healthy or not (e.g. a red or green flag at front of shop).<br>• Educate people about how to eat fruit and vegetables in more meals and more consistently.<br>• Schools and workplaces should provide people with more time for lunch so that people are not rushing and having to purchase and eat fast food.<br>• Plant fruit trees in schools and public parks and educate people about how they can grow fruits and vegetables at home.<br>• Introduce a community kitchen where people can cook together and share meals.<br>• Cooking classes that help people to discover healthy recipes for traditionally unhealthy foods e.g. how to make healthy pizzas with lots of vegetables and wholemeal bases.<br>• Promote eating together as a family or community to promote home cooking, slow eating, joy associated with food, and sharing of knowledge across generations to expose children to greater variety of meals.<br>• Teach batch cooking.<br>• Move from food banks to food clubs to help people understand what to do with food and encourage healthy eating.<br>• More culturally-appropriate and practical cooking sessions and nutrition information to ensure people can still follow traditional diets.<br>• Make sure that food bank parcels contain healthy foods that people know how to use.<br>• Local council needs to invest more consistently in their health projects and messaging.<br>• Discounted business rates for people who sell healthier options to the community.<br>• Healthier role models and trusted voices on social media who promote healthy, balanced diets not just fad diets.<br>• Promote and encourage breastfeeding.
